# The Incidence and Risk of Herpes Zoster in Patients With Sleep Disorders: A Population-Based Cohort Study

**DOI:** 10.1097/MD.0000000000002195

**Published:** 2016-03-18

**Authors:** Wei-Sheng Chung, Hsuan-Hung Lin, Nan-Cheng Cheng

**Affiliations:** From the Department of Internal Medicine, Taichung Hospital, Ministry of Health and Welfare (W-SC, N-CC); Department of Health Services Administration, China Medical University (W-SC); and Department of Healthcare Administration (W-SC) and Department of Management Information Systems (H-HL), Central Taiwan University of Science and Technology, Taichung, Taiwan.

## Abstract

Lack of sleep can compromise the immune system, which may reactivate latent varicella-zoster virus. Studies on sleep disorders and the risk of herpes zoster (HZ) are scant.

We conducted a population-based cohort study to evaluate the risk of HZ in patients with sleep disorders and potential risk factors for HZ development.

We identified patients with sleep disorders without apnea from 2002 to 2005 by using the Taiwan Longitudinal Health Insurance Database. The patients without sleep disorders were randomly selected and frequency matched with patients with sleep disorders according to age, sex, and index year. We estimated the follow-up time in person-years for the patients from the entry date until HZ diagnosis, loss to follow-up, or the end of 2010. We used Cox proportional hazards regression models and a sensitivity analysis to estimate the risk of HZ while controlling for demographic characteristics and comorbidities.

A total of 131,001 study participants (follow-up, 948,177 person-years; mean age, 51.2 ± 16.5 years; 62.2% women) were included in the study. Patients with sleep disorders exhibited a higher incidence of HZ compared with a comparison cohort when stratified by age, sex, and comorbidities. After adjustment for covariates, the sleep disorder cohort exhibited a 1.23-fold greater risk of HZ compared with the comparison cohort (95% confidence interval [CI] = 1.17–1.30). The incidence of HZ increased with age. Adults ages 65 years and older exhibited a 6.11-fold greater risk of HZ development compared with their younger counterparts (95% CI = 5.34–7.00). Cancers and autoimmune diseases were independent risk factors of HZ development.

The patients with sleep disorders may carry an increased risk of developing HZ.

## INTRODUCTION

Herpes zoster (HZ) is characterized by unilateral radicular pain and a vesicular eruption in a limited skin area caused by the reactivation of latent varicella-zoster virus (VZV).^1^ For most people with VZV, infection occurs during childhood or early adulthood.^2^ Approximately 33% of people with VZV infection develop HZ during their lifetime, and HZ incidence increases with age. Most HZ patients are ages 50 years and older.^3^ Acute HZ has a substantial impact on health-related quality of life and functional status.^4–6^ Furthermore, postherpetic neuralgia interferes considerably with life enjoyment, sleep, and general daily activity.^7,8^

Worldwide, the prevalence of sleeping problems ranges from 4% to 40%.^9^ The prevalence of sleeping problems is consistently higher in women and older people reported in 8 low-income countries across Africa and Asia.^9^ A study in Taiwan reported that over 25% of Taiwanese adults have experienced insomnia.^10^ Chronic sleep impairment can increase inflammatory markers in addition to decreasing immune function and increasing susceptibility to infection.^11,12^ Experimental studies have indicated that sleep deprivation can impair the production of antigen-specific antibodies and enhance the susceptibility to rhinovirus after delivering influenza vaccines.^13–15^

Studies have reported that the risk factors for HZ include diabetes mellitus (DM), cancers, autoimmune diseases, and human immunodeficiency virus infection and acquired immune deficiency syndrome (HIV/AIDS), which are associated with impaired cell-mediated immunity.^16–19^ No study has evaluated the relationship between sleep disorders and HZ. Therefore, we conducted a population-based cohort study to investigate the risk of HZ in patients with sleep disorders and potential risk factors for HZ development.

## METHODS

### Data Source

The Government of Taiwan launched the National Health Insurance (NHI) program in 1995. The program is a compulsory and single-buyer insurance system that has achieved providing coverage for 99% of Taiwan's residents and contracting more than 96% of healthcare institutions.^20^ The National Health Insurance Administration (NHIA) established the National Health Insurance Research Database (NHIRD) for public research. The NHIRD contains demographic and claims data on outpatient and inpatient services and drug prescriptions. In this retrospective cohort study, the Longitudinal Health Insurance Database 2000 (LHID), a subset of the NHIRD, was used as a data source. The LHID contains original claims data of 1,000,000 beneficiaries enrolled in 2000. The LHID population was randomly sampled from the Registry for Beneficiaries of the NHIRD, which contains the registration data of every beneficiary of the NHI program in 2000. To protect the personal information, patient data were encrypted and depersonalized by the NHIA before the LHID was released for public use. This database has been used reliably in numerous nationwide cohort studies.^21,22^ The patient diagnoses in the LHID are coded on the basis of the International Classification of Diseases, Ninth Revision, Clinical Modification (ICD-9-CM). This cohort study was approved by the Institutional Review Board of Tsaotun Psychiatric Center.

### Sampled Patients

We selected adults ages 20 years and older with newly identified sleep disorders (ICD-9-CM Codes 307.4 and 780.5) as the sleep disorder cohort, including nonorganic sleep disorders and sleep disturbances diagnosed by physicians from January 2002 to December 2005. We excluded patients younger than 20 years, patients who had sleep apnea syndrome (ICD-9-CM Codes 780.51, 780.53, and 780.57), or those with incomplete information regarding sex or date of birth. The date of sleep disorder diagnosis was used as the entry date for the patients with a sleep disorder. The comparison cohort comprised 1 person for each sleep disorder patient; patients without a history of sleep disorder were randomly selected and frequency-matched according to sex, age, and entry date.

### Outcome Measures

The outcome of interest was a diagnosis of HZ (ICD-9-CM Code 053) recorded in the LHID. We determined the follow-up person-years by calculating the interval from the entry date until HZ diagnosis, withdrawal from the NHI program, death, or until December 31, 2010, whichever first occurred. Furthermore, we commenced a sensitivity analysis for the outcome interest by the diagnosis of HZ and receiving antiviral medicine. Antiviral medicine includes acyclovir, famciclovir, and valacyclovir.

### Covariate Assessment

Age stratification was categorized into ≤34, 35 to 44, 45 to 54, 55 to 64, and ≥65 years. The comorbidities included cancer (ICD-9-CM Codes 140–208), DM (ICD-9-CM Code 250), autoimmune diseases (ICD-9-CM Codes 714.0, 710.0, 710.1, 710.2, 710.3, 710.4), and HIV/AIDS (ICD-9-CM Code 042).

### Statistical Analysis

All statistical analyses were performed using SPSS Version 17.0 (SPSS Inc., Chicago, IL). The proportionate distribution of demographic characteristics and comorbidities between the sleep disorder patients and the controls were compared and tested by using the chi-squared test, and the mean age of both cohorts was measured and tested by using the Student *t* test. HZ incidence rates were estimated according to the number of follow-up person-years. Overall age-, sex-, and comorbidity-specific incidence of HZ for both cohorts was assessed and univariate and multivariate Cox proportional hazard regression analyses were used to calculate the crude hazard ratios (HRs) and adjusted HR with 95% confidence intervals (CIs) of HZ development in the sleep disorder cohort. Subsequently, the results were compared with those of the cohort without sleep disorders.

The incidence rates and HRs for the interaction between sleep disorders and comorbidities on the development of HZ were also measured. The Kaplan–Meier analysis and log-rank test were applied to assess the difference in the HZ-free rates between the 2 cohorts. All tests were 2-tailed and with a significance level of *P* < 0.05.

## RESULTS

### Comparison of Demographics and Comorbidity Between Sleep Disorder Patients and Controls

The mean follow-up years of the patients with sleep disorders and the comparison cohort were 7.28 ± 1.61 and 7.41 ± 1.52 years, respectively (data not shown). Because of sex and age matching, these demographic characteristics did not differ between the 2 cohorts. The 64,548 sleep disorder patients and 66,453 controls without sleep disorders had a mean age of 51.2 ± 16.5 years and most were women (62.2%). Compared with the controls, the patients with a sleep disorder had a higher proportion of medical comorbidities, namely DM (12.3% vs 10.2%), cancer (4.3% vs 2.9%), and autoimmune diseases (2.0% vs 1.0%). We did not further analyze the HIV/AIDS cases because they were limited in frequency (Table 1).

**TABLE 1:**
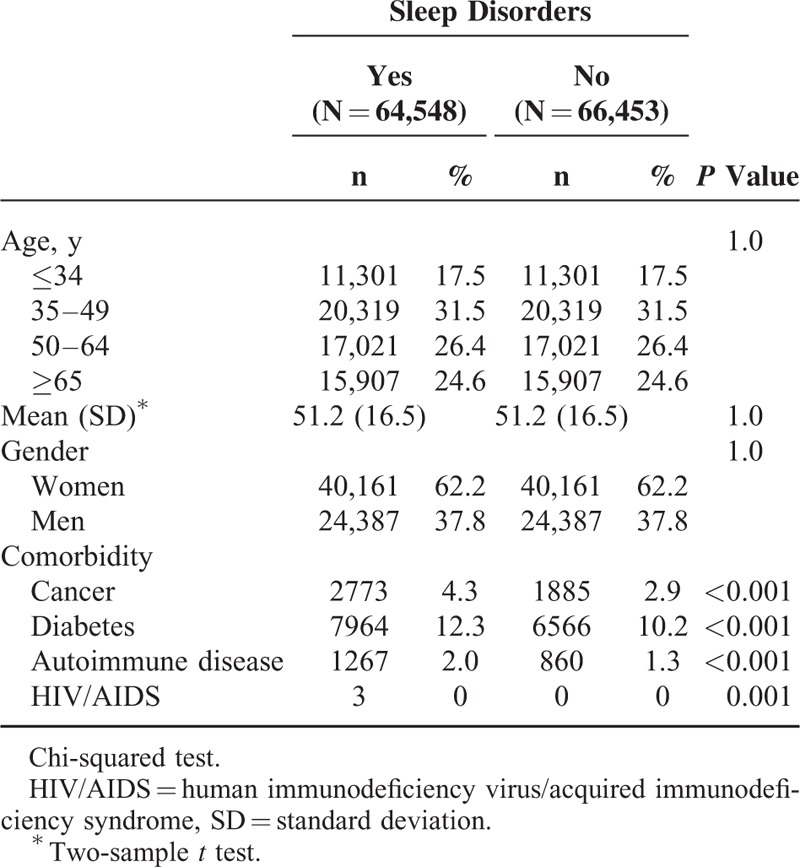
Comparison of Demographics and Comorbidity Between Patients With Sleep Disorders and Comparison Cohort

### Incidence Rate and Risk of HZ Between Sleep Disorder Patients and Controls

The incidence rates of HZ over time were higher in the patients with a sleeping disorder than in the controls (5.84 vs 4.72 per 1000 person-years). After adjustment for covariates, the patients with sleep disorders exhibited a 1.23-fold greater risk of HZ (adjusted HR = 1.23, 95% CI = 1.17–1.30), as well as higher incidence rates in all age, sex, and comorbidity groups. The incidence of HZ increased with age for both cohorts and was higher for the patients in the SD cohort. After adjustment for covariates, the risk was the highest in the adults ages 65 years and older (adjusted HR = 6.11, 95% CI = 5.34–7.00), followed in the adults ages 50 to 64 years (adjusted HR = 4.89, 95% CI = 4.27–5.60), and in the adults ages 35 to 49 years (adjusted HR = 2.24, 95% CI = 1.94–2.58) compared with those younger than 35 years. No significant difference in the HZ risk was observed between the men and women. The incidence of HZ was greater in patients with any of the comorbidities than in the patients with no comorbidity. However, cancer and autoimmune diseases were independent risk factors of HZ (adjusted HR = 1.23, 95% CI = 1.09–1.39 and adjusted HR = 1.42, 95% CI = 1.20–1.68, respectively; Table 2).

**TABLE 2:**
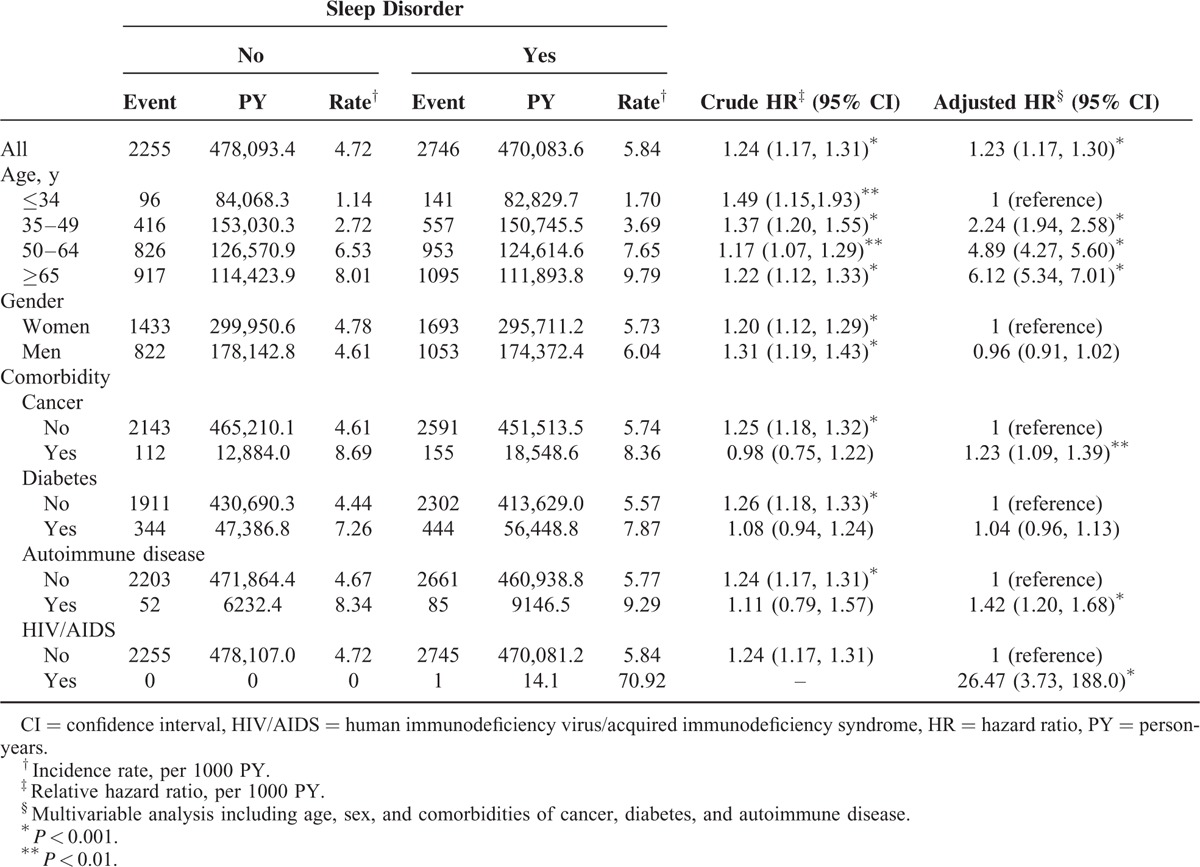
Comparison of Incidence Rate and Hazard Ratio of Herpes Zoster Between Patients With Sleep Disorders and Comparison Cohort

### Joint Effect of Sleep Disorders Associated With Cancer and Autoimmune Diseases on Developing HZ

Table 3 shows the joint effects of sleep disorders and comorbidities on developing HZ. The sleep disorder patients with cancer and autoimmune disease exhibited a substantially increased risk of HZ compared with the controls with neither autoimmune disease nor cancer.

**TABLE 3:**
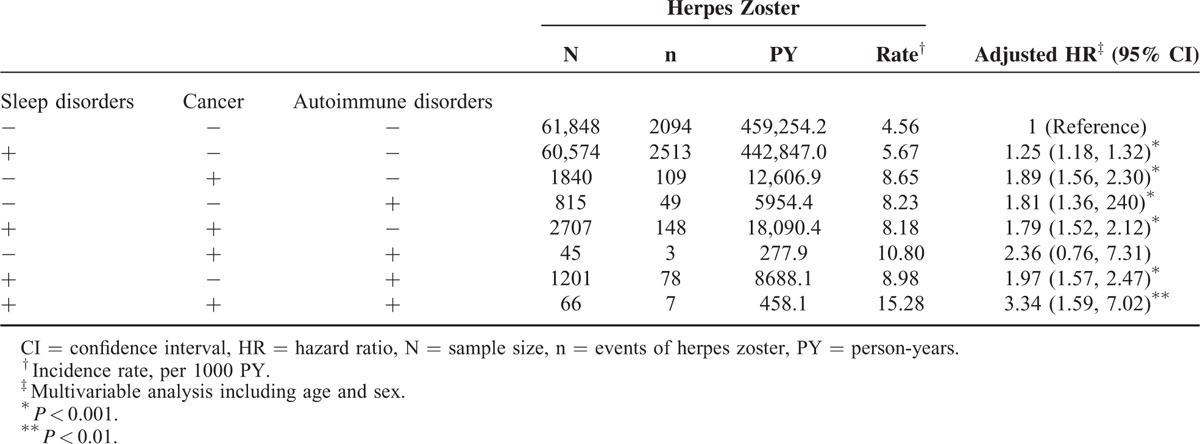
Joint Effect of Sleep Disorders Associated With Cancer and Autoimmune Disorders on the Development of Herpes Zoster

### Sensitivity Analyses Evaluating the Effect of Sleep Disorders on the Developing HZ

When considering the diagnosis of HZ and antiviral medicine, the patients with sleep disorders still exhibited a 1.34-fold increased risk of developing compared with that in the patients without sleep disorders (adjusted HR = 1.34, 95% CI = 1.22–1.47; Table 4).

**TABLE 4:**
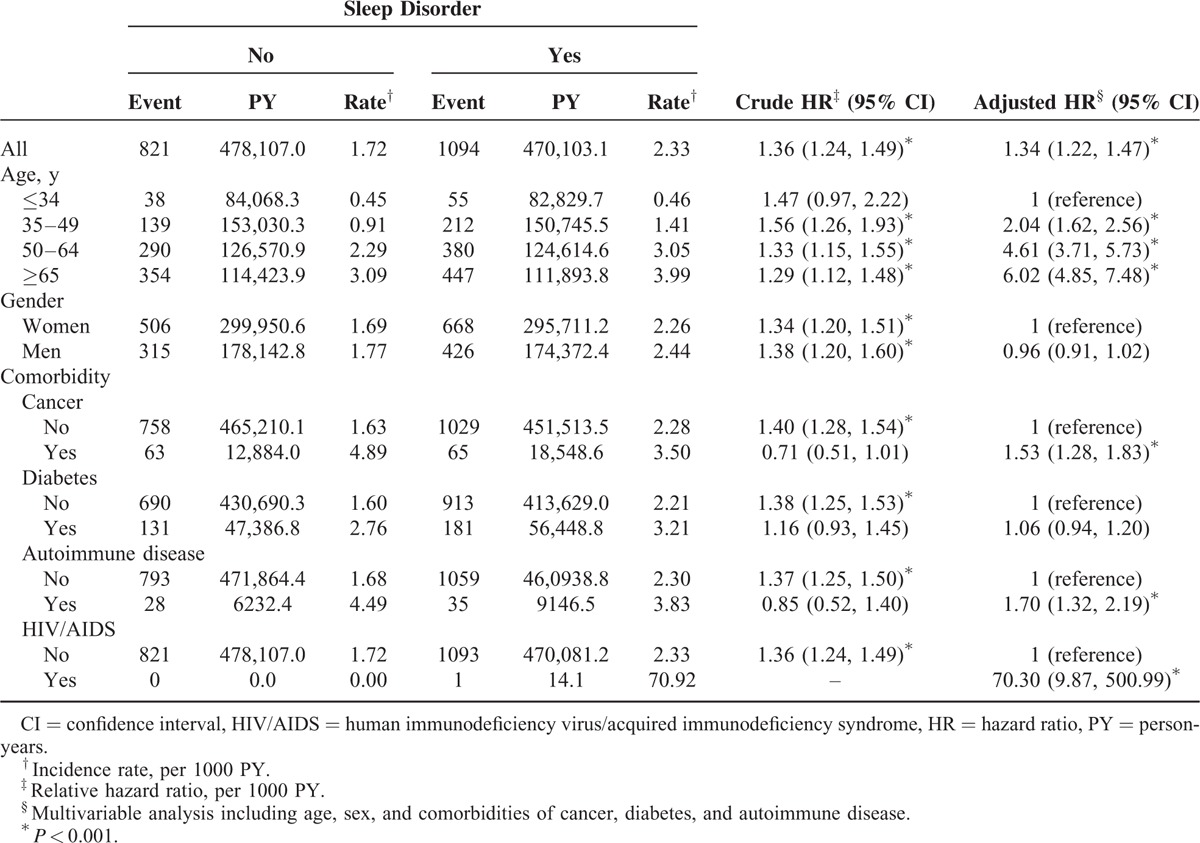
Comparison of Hazard Ratio of Herpes Zoster and Receiving Antiviral Agents Between Patients With Sleep Disorders and Comparison Cohort

### Cumulative Incidence of Being HZ-Free for the Sleep Disorder Cohort and the Cohort Without Sleep Disorders During Follow-Up Periods

**FIGURE 1:**
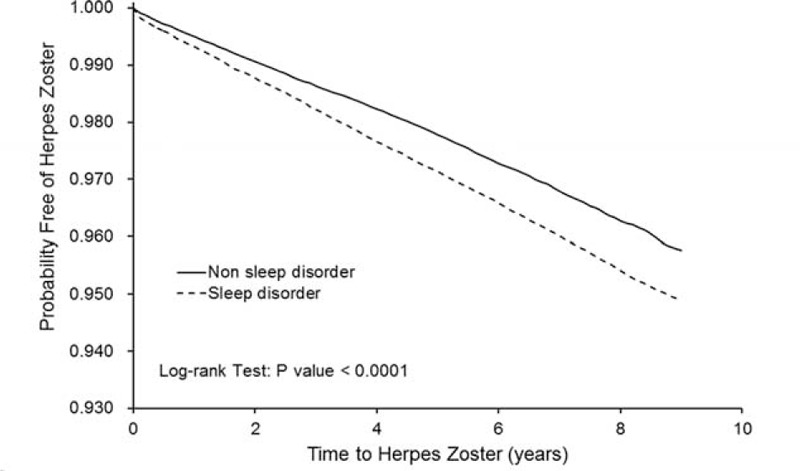
Kaplan–Meir method comparing cumulative probability free of herpes zoster between patients with sleep disorders and comparison cohort.

Figure 1 shows the Kaplan–Meier curve of HZ-free survival for the sleep disorder patients and controls. The results show that the HZ-free rate was significantly lower for sleep disorder patients compared with the controls (log-rank *P* < 0.0001).

## DISCUSSION

Based on our literature review, this is the first study to investigate the risk of HZ in patients with a sleep disorder in an Asian population. We observed a greater incidence of HZ in the sleep disorder cohort than in the comparison cohort when the cohorts were stratified by age, sex, and comorbidities. Comorbidities, namely DM, cancer, and autoimmune diseases, were also more prevalent among the patients with sleep disorders. After adjustment for potential covariates, patients with sleep disorders exhibited a 1.23-fold greater risk of HZ than did the comparison cohort.

The biological mechanism of epiphenomenons increasing the risk of HZ in the sleep disorder cohort was unclear. Following primary infection, the VZV virus remains dormant in the ganglia of sensory cranial nerves and the spinal dorsal ganglia root. Since cellular immunity decreases, the VZV virus can reactivate and travel along the ganglia and manifest as HZ on the skin. According to a seroprevalence survey in Taiwan, in up to 90% of cases, VZV infection occurs during childhood or early adulthood.^2^ The relationship between sleep and immune function is bidirectional. Sleep complaints are common in patients with HIV/AIDS,^23^ systemic lupus erythematosus,^24^ and systemic sclerosis.^25^ Moreover, sleep disorders can disrupt the immune system and increase susceptibility to infectious diseases.^26,27^ Previous studies have indicated that sleep loss can reduce immune function, including reduced killer cell activity and inhibited interleukin (IL)-2 production, and can activate certain inflammatory cytokines such as IL-1, IL-6, and tumor necrosis factor alpha.^15,28–30^ In addition, the lack of sleep can negatively affect in vivo antibody production in response to vaccination, which could explain the association of sleep disorders with the increased risk of infection.^31^

In this study, most of the patients with sleep disorders were women, which is consistent with previous studies.^9,32^ However, no significant difference of HZ development was observed between sexes. The incidence of HZ increased with age. Moreover, the adults ages 65 years and older exhibited a 6.11-fold increased risk of HZ compared with those who were younger (adjusted HR = 6.11, 95% CI = 5.34–7.00). Advanced age is a considerable risk factor for the reactivation of HZ and subsequent postherpetic neuralgia.^33^ Furthermore, postherpetic neuralgia and acute pain from HZ cause substantial impairment of functional performance and quality of life in older adults.^5,6^ Recent studies have reported that the HZ vaccine reduces HZ development and HZ-related impairment affecting activities of daily living.^34–36^ Therefore, older adults should consider receiving the HZ vaccine if it is available.

After adjustment for covariates, cancer and autoimmune diseases were independent risk factors of HZ. These findings are consistent with those in previous studies.^37^ Patients with cancers and an immune system impaired by autoimmune diseases after taking immunosuppressive agents are at an increased risk of HZ because of altered cell-mediated immunity.^38^

The strength of this study is that it provides a large population-based cohort that was derived from an Asian population to investigate the link between sleep disorders and the risk of subsequent HZ. The diagnoses of sleep disorders and HZ were made by physicians instead of by using a questionnaire survey. We were able to trace the patients throughout the follow-up period because the NHI beneficiaries are assigned personal identification numbers under the exclusive NHI program. We further conducted a sensitivity analysis by a HZ diagnosis with antiviral agents and still exhibited patients with sleep disorders were at an increased risk of developing HZ. The Taiwan NHIA routinely reviews the claims data and the medical records through administrative and peer-review process. The NHIA provides both civil and criminal penalties for healthcare fraud.

However, some limitations must be considered when interpreting these findings. First, patients with sleep disorders may have been underrepresented because patients with insomnia might not visit a physician to discuss their sleep problems.^21^ Second, the lack of detailed immunosuppressive drug information may become a potential confounding factor affecting the results. However, the medical comorbidities examined in this study are related to the use of immunomodulatory drugs, which may mediate the effect of immunomodulatory drugs. Third, the medical comorbidities are diagnosed by physicians not based upon administered medications may result in misclassification.

In summary, our population-based cohort study investigating 64,548 patients with 470,084 person-year follow-ups indicated that patients with sleep disorders are at an increased risk of subsequent HZ compared with the general population. Because the number of patients with sleep disorders is increasing, the management of sleeping problems must be improved to facilitate preventing HZ. However, this is a retrospective observation study investigating epiphenomenons associated with HZ; future studies are warranted to elucidate the biological mechanisms of sleep disorders on the effect of developing HZ.
